# Deep Neural Network Classifier for Virtual Screening Inhibitors of (S)-Adenosyl-L-Methionine (SAM)-Dependent Methyltransferase Family

**DOI:** 10.3389/fchem.2019.00324

**Published:** 2019-05-10

**Authors:** Fei Li, Xiaozhe Wan, Jing Xing, Xiaoqin Tan, Xutong Li, Yulan Wang, Jihui Zhao, Xiaolong Wu, Xiaohong Liu, Zhaojun Li, Xiaomin Luo, Wencong Lu, Mingyue Zheng

**Affiliations:** ^1^Department of Chemistry, College of Sciences, Shanghai University, Shanghai, China; ^2^State Key Laboratory of Drug Research, Drug Discovery and Design Center, Shanghai Institute of Materia Medica, Chinese Academy of Sciences, Shanghai, China; ^3^School of Pharmacy, University of Chinese Academy of Sciences, Beijing, China; ^4^Department of Pediatrics and Human Development, Michigan State University, East Lansing, MI, United States; ^5^School of Pharmacy, East China University of Science and Technology, Shanghai, China; ^6^School of Life Science and Technology, Shanghai Tech University, Shanghai, China; ^7^School of Information Management, Dezhou University, Dezhou, China

**Keywords:** deep neural network, virtual screening, methyltransferase, epigenetic, drug design

## Abstract

The (S)-adenosyl-L-methionine (SAM)-dependent methyltransferases play essential roles in post-translational modifications (PTMs) and other miscellaneous biological processes, and are implicated in the pathogenesis of various genetic disorders and cancers. Increasing efforts have been committed toward discovering novel PTM inhibitors targeting the (S)-Adenosyl-L-methionine (SAM)-binding site and the substrate-binding site of methyltransferases, among which virtual screening (VS) and structure-based drug design (SBDD) are the most frequently used strategies. Here, we report the development of a target-specific scoring model for compound VS, which predict the likelihood of the compound being a potential inhibitor for the SAM-binding pocket of a given methyltransferase. Protein-ligand interaction characterized by Fingerprinting Triplets of Interaction Pseudoatoms was used as the input feature, and a binary classifier based on deep neural networks is trained to build the scoring model. This model enhances the efficiency of the existing strategies used for discovering novel chemical modulators of methyltransferase, which is crucial for understanding and exploring the complexity of epigenetic target space.

## Introduction

Methyltransferases (MTases) are a class of enzymes that transfer methyl groups to the substrates including DNA, proteins and small molecules (Zhang and Zheng, 2016). Based on different substrates, MTases can be divided into three classes: DNA methyltransferases (DNMTs) (Da Costa et al., 2017), protein methyltransferases (PMTs) (Boriack-Sjodin and Swinger, 2016) and MTases for small molecules like catecholamines (Bonifácio et al., 2007). Most methyltransferases use S-adenosyl-L-methionine (SAM) as a donor for methyl groups, where all have a SAM-binding pocket and a substrate-binding pocket (Martin and McMillan, 2002). These SAM-dependent MTases participate in numerous essential biological processes, including the epigenetic control of cell fate, cell signaling and degration of metabolites (Hu et al., 2015; Schapira, 2016). Consequently, the dysregulation of MTases have been implicated in diverse diseases including of many types of cancers (Kaniskan et al., 2015), metabolic disorders (Deng et al., 2013), cardiovascular disease (Bouras et al., 2013), inflammatory response (Sun et al., 2015), neurological disorders (Meaney and Ferguson-Smith, 2010), and so on. Therefore, SAM-dependent MTases have been considered as a type of intriguing targets for pharmacological intervention, and interest in developing potent MTase inhibitors continues to grow in both academic laboratories and pharmaceutical companies (Hu et al., 2016). Targeting the SAM-binding pocket is an effective strategy for designing methyltransferase inhibitors, akin to targeting the ATP-binding pocket of kinases (Wu et al., 2015). A number of inhibitors binding to SAM pocket have been reported, including SGI-1027 (Rilova et al., 2014), CPI-1205 (Vaswani et al., 2016), EPZ-6438 (Kuntz et al., 2016), GSK-126 (McCabe et al., 2012), EPZ-5676 (Stein et al., 2018), and so on (Biswas and Rao, 2018). Among them, pyridone-based EZH2 inhibitors CPI-1205, EPZ-6438 and GSK-126 have been in phase I clinical trials. In addition, compound EPZ-5676 has finished phase I clinical trials for relapsed/refractory leukemias bearing a rearrangement of the MLL gene, and has modest clinical activity in adult acute leukemia. So far, there is still no small molecule MTases inhibitors being approved, and many projects were temporarily halted partially due to poor *in vivo* activity or unsatisfactory bioavailability of current chemo types. Therefore, finding of MTases inhibitors with novel scaffolds is still a challenging research area.

To discover and design new MTases inhibitors more efficiently, a variety of computational methods have been developed and used in combination with experiment methods (Kireev, 2016). For example, virtual screening based on molecular docking has been widely used to discover potential small molecule leads (Kireev, 2016). Existing molecular docking methods typically consists of conformation searching and a scoring function for complex binding affinity evaluation (Morris and Lim-Wilby, 2008). These molecular docking methods can produce the binding poses with acceptable accuracy, but they are less successful in scoring and active compound ranking, leading to high false positive rates in virtual screening campaigns (Berishvili et al., 2018). Furthermore, the performance of molecular docking for different targets may vary widely, especially with regard to the complexity of methyltransferase family targets. Previously our group constructed a knowledge-based general-purposed scoring function iPMF (Shen et al., 2011), which utilizes the interative-extracted statistical potentials from protein-ligand complexes. However, the SAM-binding sites exhibit great polarity and structural flexibility; therefore, it is difficult for the general-purpose scoring functions like iPMF to perform satisfactorily for this system. It is therefore a practical compromise constructing a scoring function specific for SAM-dependent MTases. Many target-specific scoring functions have been constructed through different methods to improve the performance of existing scoring functions on certain targets to varying degree (Xing et al., 2017; Berishvili et al., 2018). Recently, our group developed a SAM-dependent methyl transferase-specific scoring function SAM-score using ε-SVR, and used this scoring function in discovery of a new class of DOT1L inhibitors (Wang et al., 2017). Regrettably, despite a lower rate of false positive in our in-house use, the SAM-score still leaves large room for improvement. For example, the Enrichment Factor (EF) (5%) of SAM-score was only 1.46 in one of our recent tests, which means that the screening power of the scoring model is not satisfactory.

Recently, deep learning-based approaches have emerged in the field of scoring function. For instance, Jiménez et al. constructed a general-purpose scoring function K_DEEP_ via 3D-convolutional neural networks (Jiménez et al., 2018). There are clear differences between deep learning and traditional machine learning methods, for example: traditional machine learning methods uses sparse representations to describe the input data, and learning-task related features are further extracted from the representations, which needs extensive domain knowledge and time investment, and may lose some important information in the process; while the representation learning framework of deep learning methods uses distributed representations for the dataset and then automatically extract features, which can extract abstract higher-level features and finally generate more accurate prediction results (LeCun et al., 2015).

In this study, we developed a SAM-dependent MTases-specific classifier based on a fully connected neural network to accurately distinguish between negative (inactive) and positive (active) MTases inhibitors. First, crystal structures of the SAM-dependent MTases and the compounds with experimental affinity data against these targets were collected. Decoys for each targets were also generated to expand the data set in this step. Then, molecular docking was used to produce protein-ligand interaction conformations. Here, the Fingerprinting Triplets of Interaction Pseudo atoms (TIFP) (Desaphy et al., 2013) were used to describe the predicted complex conformations. In the next step, these TIFPs were used as inputs to establish a fully connected neural network model by mining the structure and activity relationship of previously reported small molecules for different MTases. The performance of the DNN model were also compared with Glide, Autodock·vina, and the mixed model of DNN and Glide. The results showed that DNN model can significantly improve the screening power of docking and has the ability to prioritize active molecules with diverse scaffolds. Moreover, this model can also help to determine the selectivity of the compounds targeting different MTases, which may provide insight into developing novel inhibitors of SAM-dependent MTases.

## Results and Discussion

**Figure 1:**
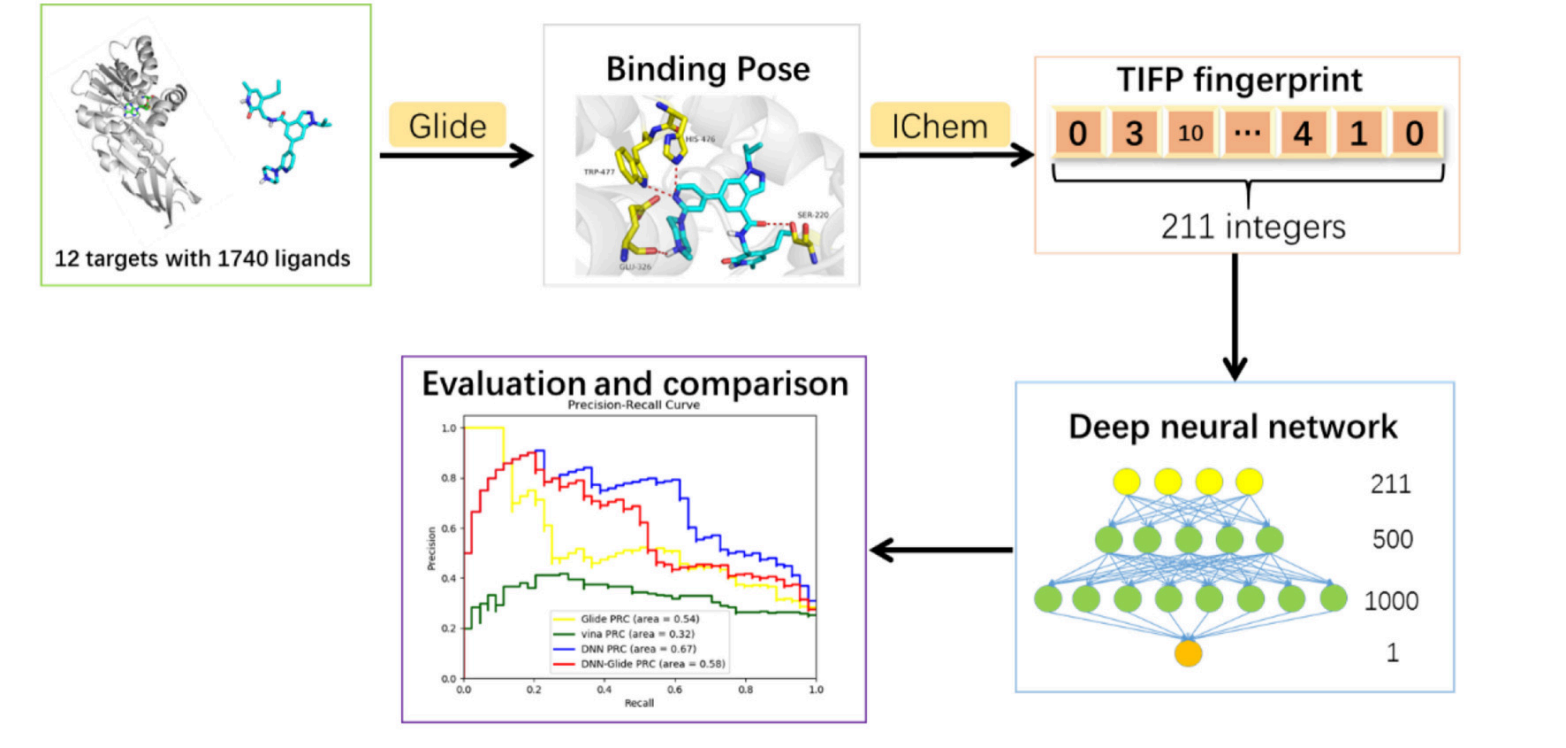
Overall workflow of model construction.

This research was aimed to build a target-specific classification model to distinguish whether a compound is a potential inhibitor of a given methyltransferase. The workflow contains deep neural network model construction and model evaluation steps, which will be explained in details below. The overall workflow of this study was shown in Figure 1.

### Deep Neural Network Model Construction

#### Data Sources

**Figure 2:**
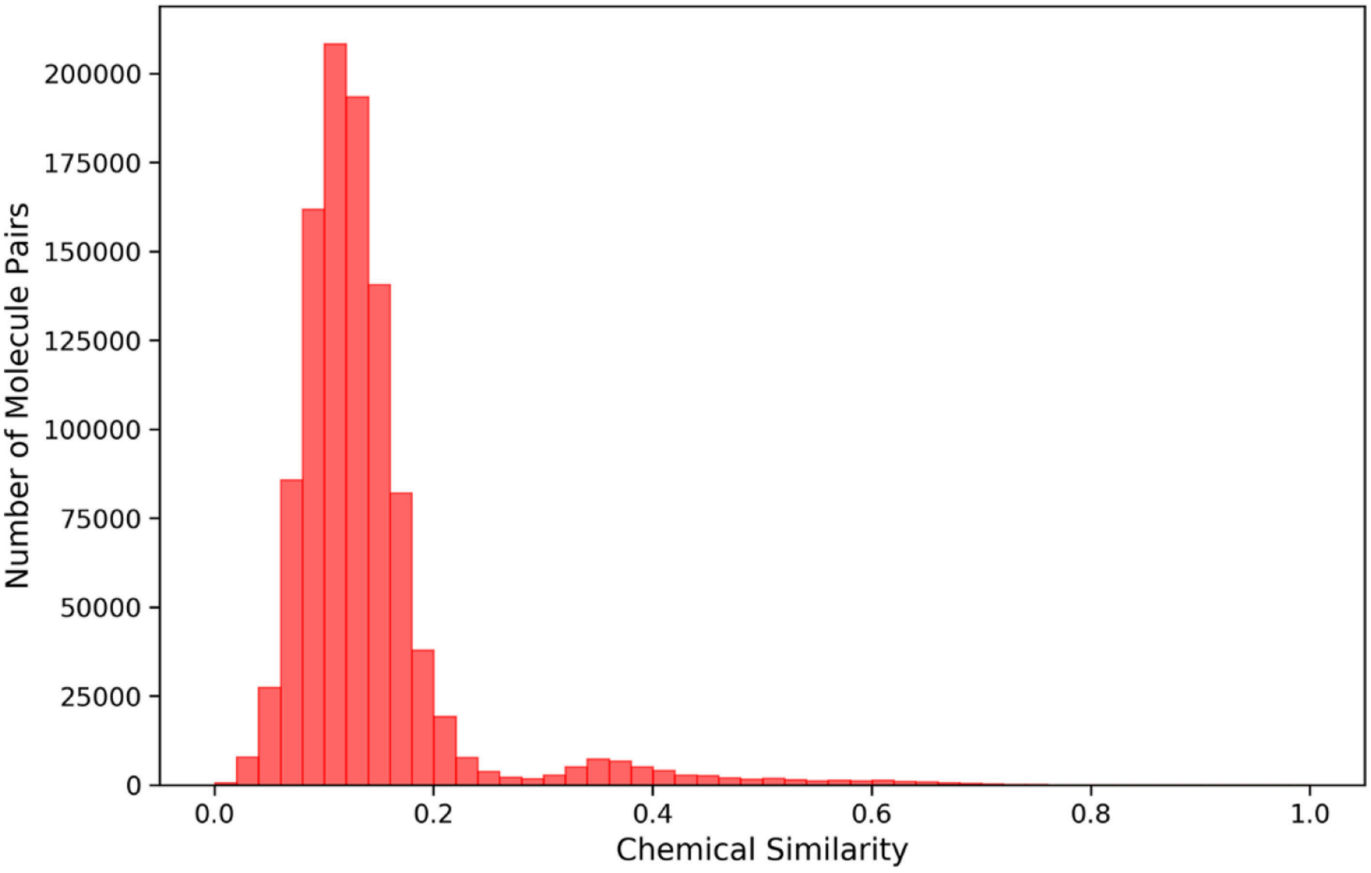
Histogram showing the distribution of chemical similarity of any two molecules in the dataset.

Based on the previous work of our workgroup, the data used to build model include the same set of 12 SAM-dependent methyltransferases, which are DNA (cytosine-5)-methyltransferase 1 (DNMT1), coactivator-associated arginine methyltransferase 1 (CARM1), protein arginine N-methyltransferase 1 (PRMT1), protein arginine N-methyltransferase 3 (PRMT3), protein arginine N-methyltransferase 5 (PRMT5), protein arginine N-methyl-transferase 6 (PRMT6), euchromatic histone-lysine N-methyl-transferase 1 (EHMT1), euchromatic histone-lysine N-methyltransferase 2 (EHMT2), SET domain containing lysine methyltransferase 7 (SETD7), SET domain containing lysine methyltransferase 8 (SETD8), suppressor of variegation 3-9 homolog 2 (SUV39H2) and disruptor of telomeric silencing 1-like histone H3K79 methyltransferase (DOT1L). The crystal structures in the data set are derived from the Protein Data Bank (PDB) (https://www.rcsb.org), which are all complex crystal structures with a ligand occupying the SAM pocket. The structures and activities data of small molecule ligands for the 12 targets were collected from the ChEMBL database, and the IC50, EC50, and Ki values less than or equal to 10 micromole were used as positive data, and that more than 50 micromole as negative data. Totally, there were 919 positive samples and 366 negative samples. The IC_50_, EC_50_, and K_i_ values in the activity data were normalized to PIC_50_, PEC_50_ or PK_i_ (PActivition = 9 – lg(Activation)). Furthermore, a total of 1212 decoys were generated in the DUD-E website (http://dude.docking.org/generate) (Mysinger et al., 2012) to better correspond to the fact of actual virtual screening where the negative data are much more than the positive data. Each molecule, either positive or negative, has at least one of 12 Mtase targets reported. The 211-bit TIFP interaction fingerprints (Desaphy et al., 2013) were used as inputs to construct the deep neural network classification model, due to its capability in characterizing directional molecular interactions such as hydrogen bonding and pi-pi stacking. Totally, 1740 molecules were compiled for deriving interaction features, which including 446 positive data and 1294 negative data. Tanimoto coefficients of Morgan fingerprints of any two molecules in the data set were calculated by RDKit python package (Figure 2), and most of them were below 0.2, suggesting that the data set has diverse chemical structures and would make the DNN model less biased.

#### Datasets Partition

1. The total 1,740 samples were randomly divided into two parts with the proportion 1:10, in which the smaller one was used as a test set.
2. The bigger one was shuffled and randomly divided into a validation set and a train set with the proportion of 1:8, which were used in the hyperparameter optimization processing.
3. This process of step 2 was repeated for ten times to obtain ten different training/validation datasets, and the best model among the models trained on the ten datasets was evaluated with the test set.

#### Hyperparameter Optimization

**Figure 3:**
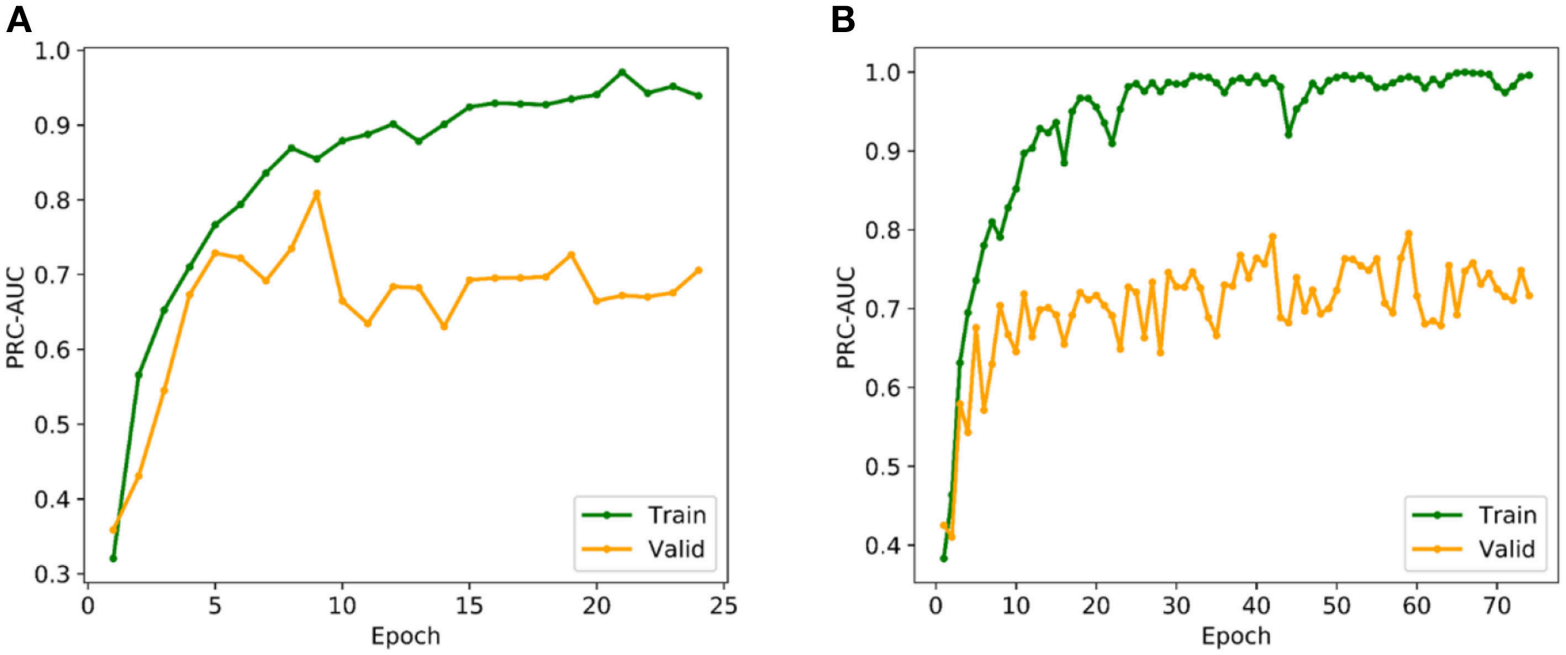
**(A)** Variation tendency of PRC-AUC with epochs in DNN model. **(B)** Variation tendency of PRC-AUC with epochs in DNN-Glide model. The PRC-AUCs of the DNN model have reached the peak on the 9th epoch, while the DNN-Glide reached the peak on the 59th epoch.

The multi-grid searching method was applied to the optimization of the hyperparameters. Because the area under Precision-Recall curve (PRC-AUC) is more informative than the area under receiver operating characteristic curve (ROC-AUC) when evaluating classifiers on imbalanced datasets (Saito and Rehmsmeier, 2015), PRC-AUC on the validation set was used for the evaluation of the hyperparameters. During training process, Adam optimizer was used for model optimization and cross-entropy was utilized as the loss function, which is a common loss function for classification model. Early stopping with a stop window size of 15 was used to save training time and to prevent over-fitting, i.e., training would be stopped if the PRC-AUC on the validation set did not increase for 15 consecutive epochs. The performance of evaluated hyperparameters in the hyperparametric search are shown in Table 1. According to the best set of hyperparameters, a fully connected three-layer neural network model with two hidden layers (500 × 1,000) was established. The input layer had 211 neurons, and the output layer was softmax-standardized dichotomous probability. Learning rate, weight decay penalty and dropout were set to 0.001, 0.0001, and 0.1, respectively. The activation function was set as ReLU. Figure 3A shows the variation tendency of PRC-AUC with epochs on training set and validation set when the DNN model was trained with the best set of hyperparameters. The PRC-AUCs of DNN model have reached the peak on the ninth epoch, and the model at that epoch was used for further evaluation.

**Table 1:** The searched hyperparameters and their performance.

**Hyperparameters**				**Performance**							
				**Train**				**Valid**			
**Dropout**	**Learning rate**	**Layer size**	**Stop epoch**	**Recall**	**Precision**	**ROC-AUC**	**PRC-AUC**	**Recall**	**Precision**	**ROC-AUC**	**PRC-AUC**
0.1	0.0001	[500, 100]	42	0.75	0.86	0.96	0.91	0.55	0.76	0.82	0.76
0.2	0.0001	[500, 100]	46	0.68	0.67	0.90	0.79	0.57	0.68	0.80	0.72
0.1	0.0001	[100, 500]	50	0.74	0.92	0.97	0.93	0.58	0.74	0.84	0.75
0.2	0.0001	[100, 500]	40	0.59	0.94	0.94	0.88	0.42	0.88	0.81	0.75
0.1	0.0001	[320, 640]	31	0.68	0.93	0.97	0.92	0.47	0.86	0.81	0.76
0.2	0.0001	[320, 640]	40	0.69	0.91	0.96	0.91	0.47	0.86	0.81	0.74
0.1	0.0001	[500, 1,000]	29	0.79	0.87	0.97	0.91	0.58	0.70	0.84	0.76
0.2	0.0001	[500, 1,000]	22	0.42	0.94	0.86	0.77	0.28	0.94	0.75	0.71
0.1	0.001	[500, 100]	29	0.80	0.88	0.98	0.94	0.60	0.70	0.82	0.76
0.2	0.001	[500, 100]	27	0.54	0.84	0.92	0.82	0.49	0.70	0.82	0.74
0.1	0.001	[100, 500]	21	0.66	0.93	0.95	0.91	0.51	0.79	0.82	0.72
0.2	0.001	[100, 500]	78	0.79	0.93	0.98	0.95	0.55	0.78	0.80	0.75
0.1	0.001	[320, 640]	32	0.91	0.90	0.99	0.97	0.60	0.68	0.80	0.74
0.2	0.001	[320, 640]	102	0.83	0.99	0.99	0.98	0.58	0.84	0.81	0.72
0.1	0.001	[500, 1,000]	9	0.74	0.77	0.94	0.85	0.66	0.73	0.87	0.81
0.2	0.001	[500, 1,000]	34	0.85	0.99	0.99	0.98	0.60	0.78	0.83	0.78
0.1	0.0005	[500, 100]	18	0.67	0.80	0.93	0.85	0.60	0.78	0.79	0.74
0.2	0.0005	[500, 100]	21	0.67	0.73	0.92	0.82	0.60	0.73	0.81	0.75
0.1	0.0005	[100, 500]	43	0.88	0.96	0.99	0.99	0.62	0.83	0.83	0.77
0.2	0.0005	[100, 500]	51	0.84	0.93	0.98	0.96	0.57	0.79	0.84	0.77
0.1	0.0005	[320, 640]	28	0.82	0.96	0.99	0.98	0.51	0.71	0.81	0.74
0.2	0.0005	[320, 640]	24	0.77	0.93	0.97	0.94	0.49	0.68	0.80	0.74
0.1	0.0005	[500, 1,000]	17	0.79	0.82	0.95	0.88	0.60	0.65	0.78	0.70
0.2	0.0005	[500, 1,000]	14	0.74	0.84	0.95	0.87	0.60	0.73	0.80	0.74
0.1	0.00005	[500, 100]	82	0.72	0.92	0.97	0.93	0.53	0.78	0.84	0.77
0.2	0.00005	[500, 100]	131	0.69	0.97	0.97	0.94	0.49	0.81	0.83	0.76
0.1	0.00005	[100, 500]	55	0.51	0.94	0.92	0.84	0.36	0.83	0.78	0.72
0.2	0.00005	[100, 500]	87	0.57	0.97	0.95	0.89	0.42	0.92	0.79	0.75
0.1	0.00005	[320, 640]	40	0.57	0.95	0.93	0.87	0.42	0.88	0.77	0.73
0.2	0.00005	[320, 640]	50	0.64	0.83	0.92	0.84	0.43	0.68	0.82	0.71
0.1	0.00005	[500, 1,000]	46	0.68	0.96	0.96	0.93	0.45	0.96	0.81	0.79
0.2	0.00005	[500, 1,000]	71	0.77	0.91	0.97	0.93	0.51	0.75	0.81	0.73

### Model Evaluation and Comparison

#### DNN Model Evaluation

To validate the feasibility and effectiveness of the models, the searched best set of hyperparameters were then trained on 10 datasets and evaluated on the validation set. The performances of these 10 models were similar, as shown in Table 2, among which the performance of 2nd model has the best PRC-AUC and ROC-AUC (Bradley, 1997) here, which was selected for further evaluation on the test set. It showed PRC-AUC, ROC-AUC and EF (5%) of 0.67, 0.86 and 3.46, respectively, on the test set.

**Table 2:** The performances of 10 trained models on the validation set.

**Model**	**Recall**	**Precision**	**Accuracy**	**ROC-AUC**	**PRC-AUC**
1	0.622	0.742	0.874	0.853	0.689
2	0.800	0.653	0.856	0.876	0.793
3	0.725	0.518	0.782	0.849	0.688
4	0.638	0.750	0.845	0.822	0.746
5	0.738	0.660	0.845	0.856	0.791
6	0.682	0.612	0.810	0.863	0.785
7	0.718	0.718	0.874	0.859	0.760
8	0.547	0.690	0.787	0.817	0.723
9	0.436	0.750	0.776	0.800	0.681
10	0.535	0.767	0.845	0.813	0.714
Average	0.64 ± 0.09	0.69 ± 0.06	0.83 ± 0.03	0.84 ± 0.02	0.74 ± 0.04

**Figure 4:**
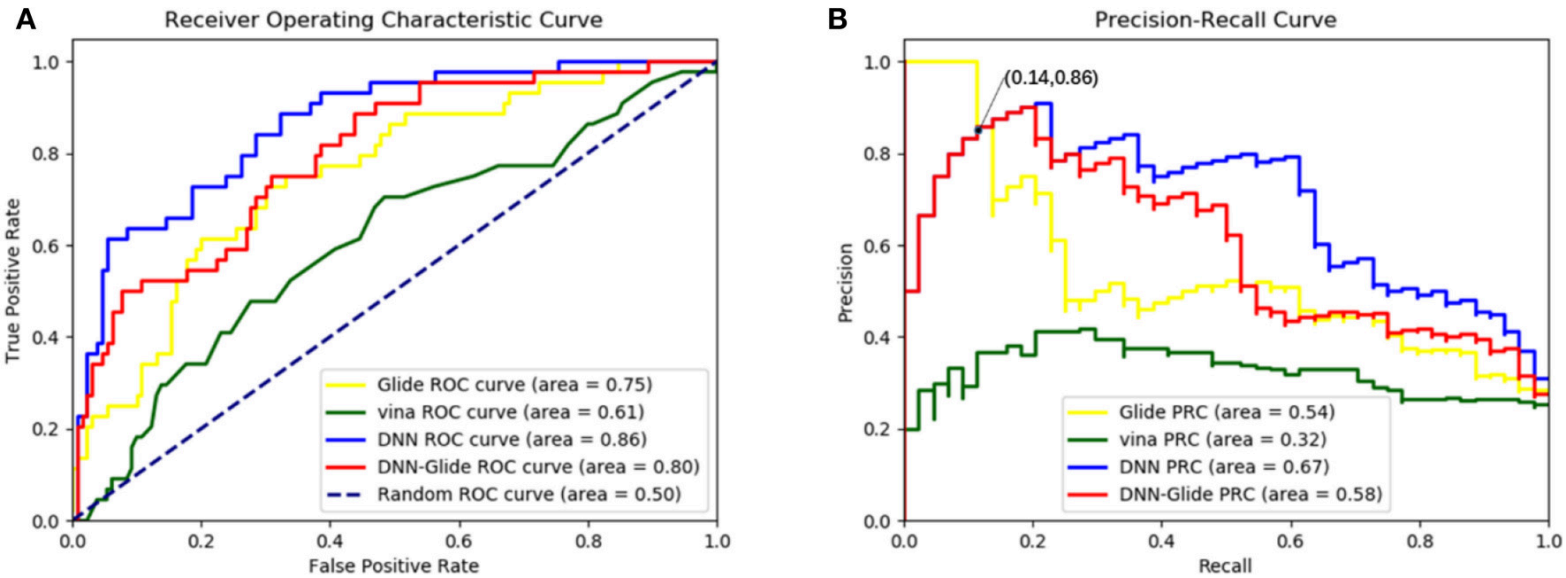
**(A)**The ROC curves of Glide, Autodock vina, DNN model and DNN-Glide model on the test set. **(B)** The PRC curves of Glide, Autodock vina, DNN model and DNN-Glide model on the test set.

**Figure 5:**
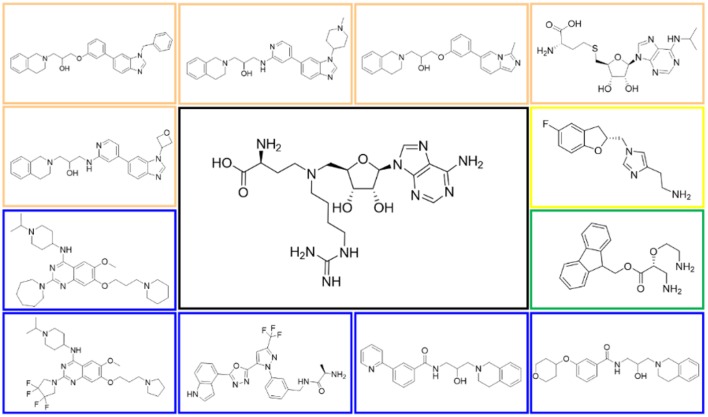
Structures of the positive compounds predicted by Glide and DNN model before the intersection. Structures in darkorange and yellow box are predicted to be positive by Glide; structures in dark, blue and green box are predicted to be positive by DNN model.

In order to evaluate the DNN model comprehensively, Glide and Autodock vina were compared with the DNN model. The docking score of the Glide SP was added as a descriptor to the end of interaction fingerprint, which was used to build a hybrid model named DNN-Glide. The DNN-Glide model was trained in the same way as DNN model and on the same datasets, and it obtained the same set of best hyperparameters as DNN model, although there is a delay of reaching the best PRC-AUC on the validation set (Figure 3B). By comparison, both the ROC curves and PRC curves of the DNN model were above that of the other models, indicating the high-quality performance of the DNN model (Figure 4 and Table 3). Especially, the true positive rate of DNN is consistently higher than that of Glide and Autodock vina when the false positive rate was extremely low, which is an obvious merit for applications in virtual screening. Unfortunately, the added Glide SP didn't improve the performance of the DNN model. It is noteworthy that the PRC curve of the Glide and DNN model intersected each other at (0.14, 0.86), before the point (Recall <0.14), the precision of Glide is higher than the DNN model. Figure 5 shows the structures of the positive compounds predicted by Glide and DNN model before the intersection point. We may find that Glide tends to retrieve compounds with one or two common scaffolds, while the DNN model is able to provide more diverse scaffolds, suggesting its generalization ability on recognizing active compounds.

**Table 3:** The performances of 4 methods on the test set, and the best performed method and its metrics are shown in bold.

**Method**	**ROC-AUC**	**PRC-AUC**	**EF (5%)**
Glide	0.75	0.54	2.97
Autodock vina	0.61	0.32	0.99
**DNN**	**0.86**	**0.67**	**3.46**
DNN-Glide	0.80	0.58	3.46

**Figure 6:**
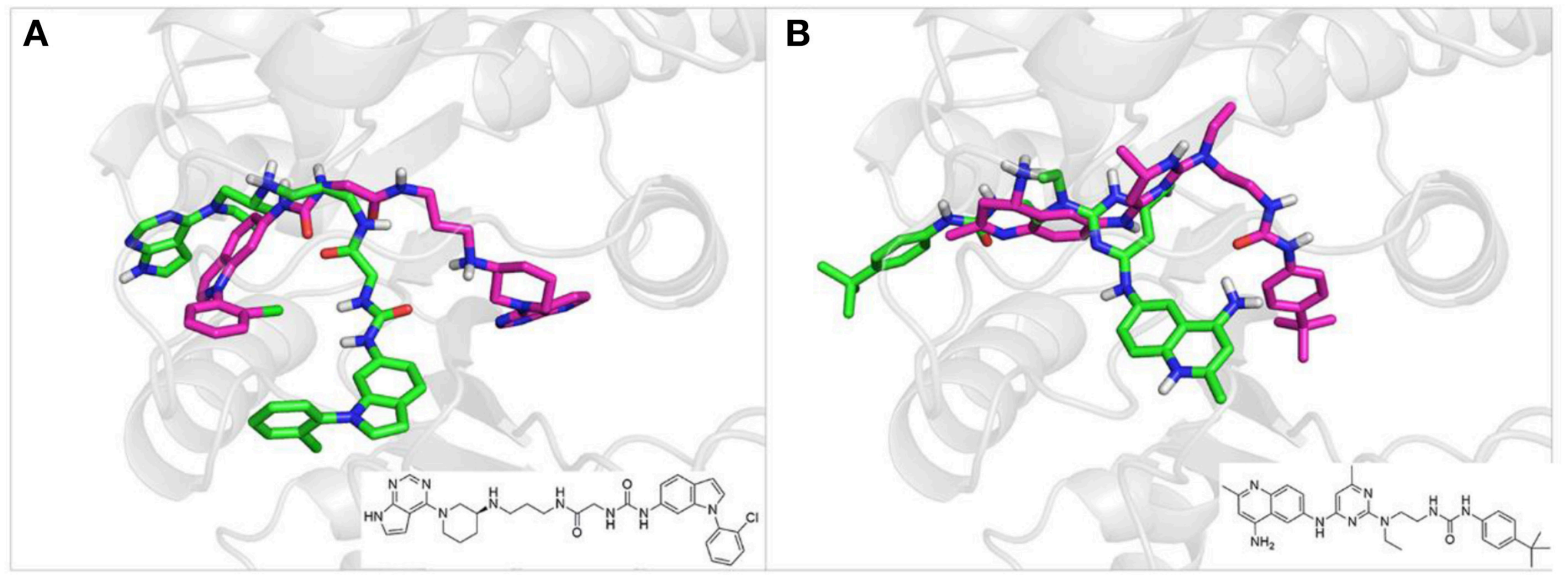
**(A)** Docking poses of the molecule “C170214_4” in PDB entry of 1NW3 (green) and 5MVS (magentas). **(B)** Docking poses of the molecule “C180722_8h” in PDB entry of 1NW3 (green) and 5MVS (magentas).

To investigate the performance of the DNN model on a specific target, an external test set containing 25 molecules was collected, which were reported binding to SAM pocket of DOT1L recently (Möbitz et al., 2017; Wang et al., 2017; Song et al., 2018). The structures and the DNN model scores of the molecules were shown in the Table 4. There are two molecules “C180722_8h” and “C170214_4” predicted far lower than the threshold of 0.5, which means that they were wrongly classified. The reason of the wrong judge was considered to be improper inputs originated from inaccurate simulated binding conformations. Since the structure of DOT1L is flexible, especially in SAM-pocket region, crystal structures obtained from experiment are quite different, which leads to various simulated binding conformations in docking (Figure 6), and different conformations may cause different results. To prove the guess, a different PDB entry 5MVS (the previous used one was 1NW3) was used as receptor structure to generate input data with the two compounds. As expected, the C170214_4 and C180722_8h was evaluated with high scores of 0.90 and 0.89, respectively, which suggests that it is vital to select a suitable receptor structure for more accurate results.

**Table 4:** The ligands of DOT1L and their scores valued by Glide, Autodock vina and DNN model.

**Label**	**Structure**	**Smiles**	**pIC_**50**_**	**Glide score**	**Vina score**	**DNN score**
C170206_10	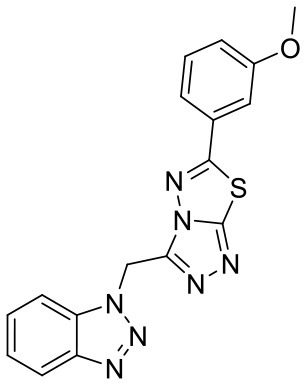	COC1 = CC = CC(= C1)C1 = NN2C(CN3N = NC4 = CC = CC = C34) = NN = C 2S1	5.19	−7.627	−8.5	0.8542
C170206_15	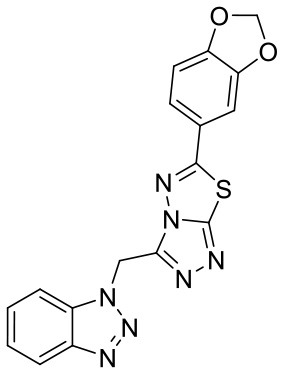	C(N1N = NC2 = CC = CC = C1 2)C1 = NN = C2 SC(= NN12)C 1 = CC = C2OC OC2 = C1	5.40	−7.915	−8.4	0.9927
C170206_16	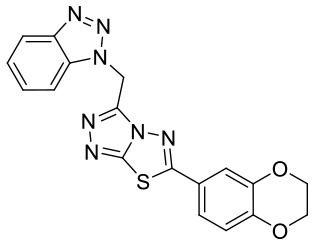	C(N1N = NC2 = CC = CC = C1 2)C1 = NN = C2SC(= NN12)C 1 = CC = C2OC COC2 = C1	5.08	−7.898	−9.9	0.9821
C170206_17	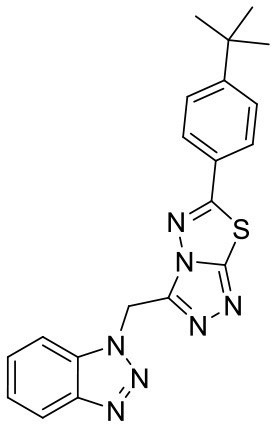	CC(C)(C)C1 = CC = C(C = C1) C1 = NN2C(C N3N = NC4 = C C = CC = C34) = NN = C2S1	5.35	−5.374	−8.8	0.555
C170206_39	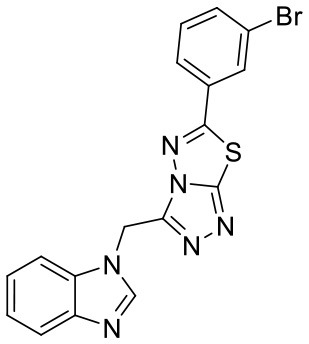	BrC1 = CC (= CC = C1)C1 = NN2C(CN3C = NC4 = CC = C C = C34) = NN = C2S1	5.39	−8.233	−8.8	0.9007
C170206_6	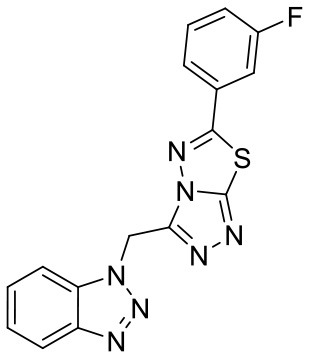	FC1 = CC = CC (= C1)C1 = NN2 C(CN3N = NC 4 = CC = CC = C 34) = NN = C2S1	5.08	−6.629	−8.7	0.8713
C170206_9	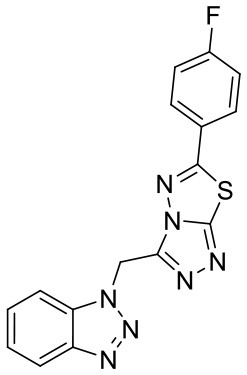	FC1 = CC = C(C = C1)C1 = NN2 C(CN3N = NC 4 = CC = C C = C34) = N N = C2S1	5.33	−7.839	−8.4	0.9439
C170214_3	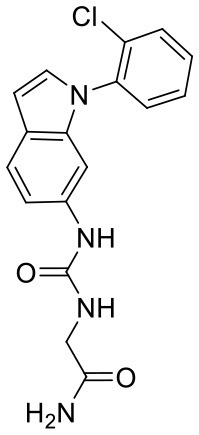	NC(= O)CN C(= O)NC1 = C C2 = C(C = C N2C2 = C(Cl) C = CC = C2) C = C1	5.05	−8.322	−8.3	0.6092
C170214_4	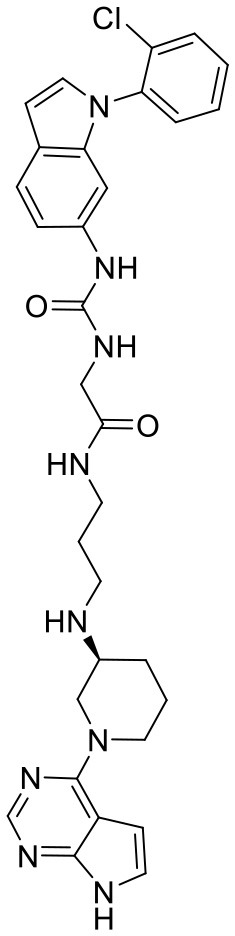	ClC1 = CC = CC = C1N1C = CC2 = C1C = C(NC(= O) NCC(= O)NC CCN[C@@H]1CC CN(C1)C1 = C3C = CNC3 = NC = N 1)C = C2	8.4	−9.432	−8.3	0.0141
C170214_5	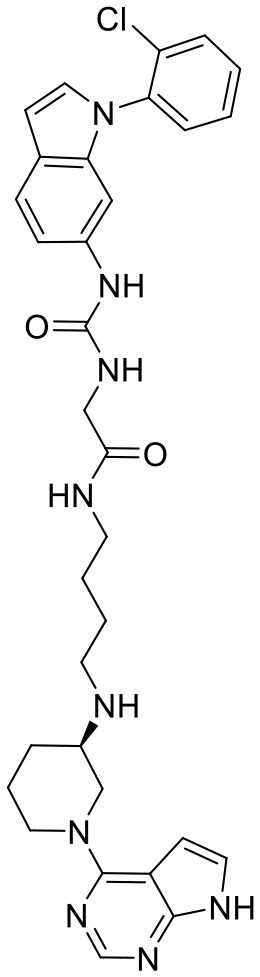	ClC1 = CC = C C = C1N1C = C C2 = C1C = C (NC(= O)NCC (= O)NCCC CN[C@@H]1CCCN (C1)C1 = C3C = C NC3 = NC = N1) C = C2	8.4	−7.773	−9.6	0.8417
C170214_6	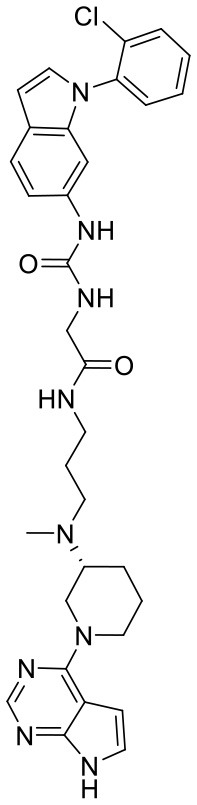	CN(CCCNC (= O)CNC(= O)NC1 = CC2 = C(C = C N2C2 = C C = CC = C2 Cl)C = C1)[C @@H]1CCCN(C1) C1 = C2C = C NC2 = NC = N1	9.82	−10.445	−9.2	0.9239
C170214_7	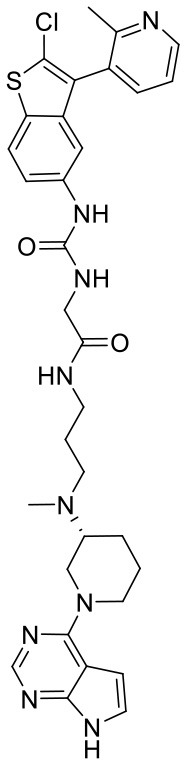	CN(CCCNC(= O) CNC(= O)NC1 = C C = C2SC(Cl) = C (C2 = C1)C1 = C C = CN = C1C) [C@@H]1CCC N(C1)C1 = C2 C = CNC2 = N C = N1	8.52	−9.125	−8.8	0.9996
C180224_6	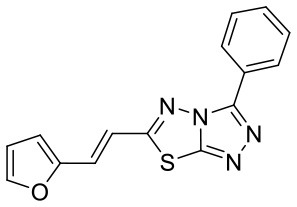	O1C = CC = C 1C = CC1 = N N2C(S1) = NN = C 2C1 = CC = CC = C1	5.15	−5.67	−7.9	0.8878
C180224_7	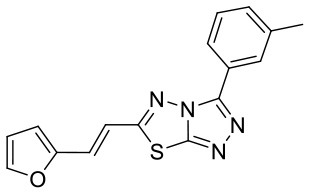	CC1 = CC = C C(= C1)C1 = NN = C2SC(C = CC3 = CC = CO3) = NN12	5.03	−7.471	−8.3	0.6535
C180224_9	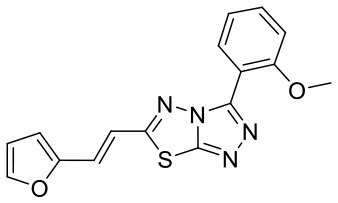	COC1 = C(C = C C = C1)C1 = NN = C2SC(C = CC3 = CC = CO3) = NN12	5.01	−6.375	−7.8	0.9412
C180722_3a	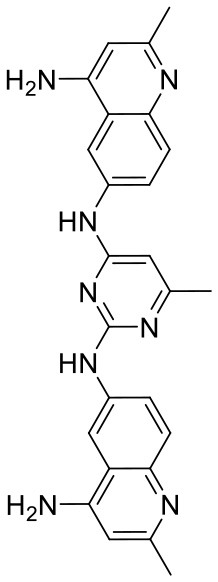	CC1 = CC(N) = C2 C = C(NC3 = NC (NC4 = CC = C5N = C(C)C = C(N)C5 = C4) = CC(C) = N3)C = C C2 = N1	5.82	−6.461	−9.8	0.7206
C180722_3b	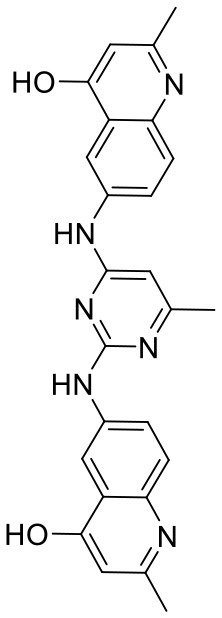	CC1 = NC(NC2 = CC = C3N = C(C)C = C(O) C3 = C2) = NC (NC2 = CC = C3N = C(C)C = C(O)C3 = C 2) = C1	5.36	−7.745	−9.1	0.8655
C180722_3d	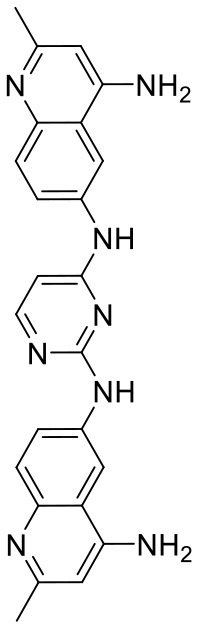	CC1 = NC2 = C C = C(NC3 = C C = NC(NC4 = C C = C5N = C (C)C = C(N)C5 = C4) = N3)C = C2C (N) = C1	5.97	−8.945	−10.1	0.7807
C180722_3e	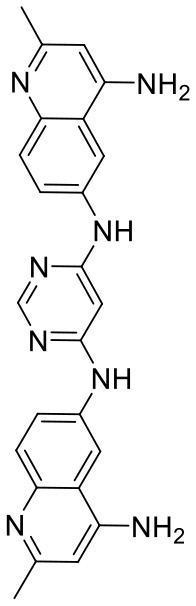	CC1 = NC2 = C C = C(NC3 = CC (NC4 = CC = C 5N = C(C)C = C(N)C5 = C4) = NC = N3) C = C2C (N) = C1	5.97	−6.465	−9.3	0.7952
C180722_8b	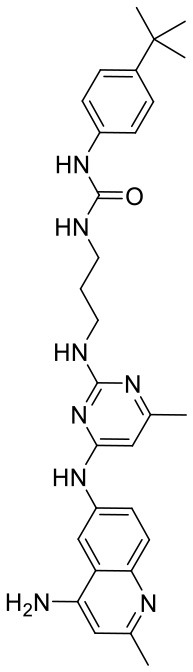	CC1 = NC(NCCCN C(= O)NC2 = C C = C(C = C2) C(C)(C)C) = NC (NC2 = CC = C 3N = C(C)C = C (N)C3 = C2) = C1	5.11	−5.558	−8.3	0.9847
C180722_8f	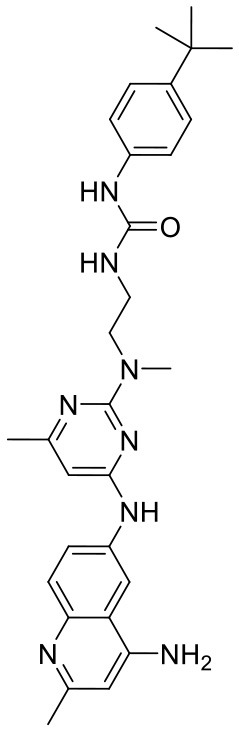	CN(CCNC(= O)NC 1 = CC = C (C = C1)C(C) (C)C)C1 = NC(NC2 = CC = C3N = C (C)C = C(N)C3 = C2) = CC(C) = N1	5.22	−7.641	−9.9	0.5408
C180722_8h	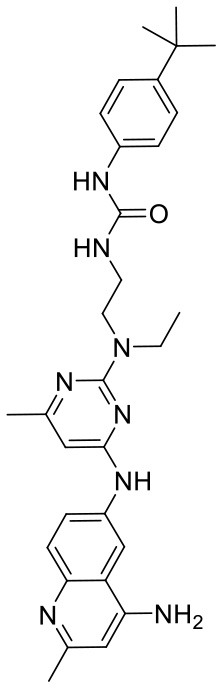	CCN(CCNC(= O)N C1 = CC = C(C = C1)C(C)(C) C)C1 = NC(NC 2 = CC = C3 N = C(C)C = C (N)C3 = C2) = CC(C) = N1	5.24	−5.082	−8.6	0.2014
C180722_8i	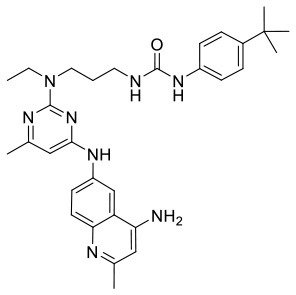	CCN(CCCNC(= O) NC1 = C C = C(C = C 1)C(C)(C)C)C 1 = NC(NC 2 = CC = C3 N = C(C)C = C (N)C3 = C2) = CC(C) = N1	5.1	−5.542	−9	0.9797
C180722_9b	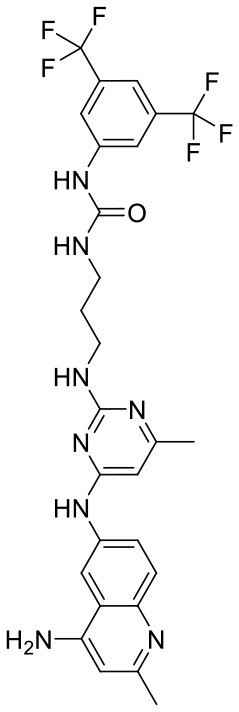	CC1 = NC(NCC CNC(= O)NC 2 = CC(= CC(= C2) C(F)(F)F)C (F)(F)F) = NC(NC2 = CC = C3N = C(C) C = C(N)C3 = C2) = C1	5.06	−7.433	−8.7	0.9993
C180722_9e	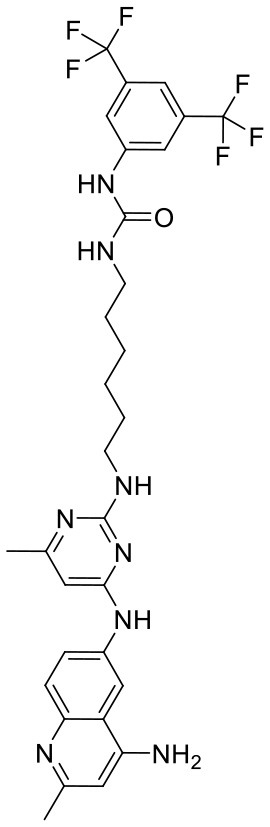	CC1 = NC(NCC CCCCNC(= O)NC2 = CC(= CC(= C2)C(F) (F)F)C(F)(F)F) = NC(NC2 = CC = C3N = C(C)C = C(N)C3 = C2) = C1	5.45	−7.664	−9.3	0.9985

### Methods

#### Ligand-Protein Binding Conformations Generation

Accurate binding poses of protein-ligand complexes are required for extracting interaction information. In view of the fact that most collected molecules don't have available complex crystal structures with their related target, we used molecular docking to produce the binding conformations.

Cross docking was carried out to choose an appropriate receptor structure of each target for generating binding poses. At first, all the complex crystal structures of each target were aligned via pymol software (version 1.8.2.2) (Schrodinger, 2015), and then the Xglide module of Maestro version 10.2 (Schrödinger, LLC, New York, NY, 2015-2) was used for cross docking. In this process, every ligand extracted from a crystal structure was docked to collected crystal structures of the target, and the root-mean-square deviation (RMSD) values of the docked poses with reference to the corresponding native poses in the crystal structures were calculated. For every target, the crystal structure with the smallest average RMSD of all extracted ligands of this target was selected as the receptor structure for the next molecular docking. Selected protein crystal structure structures and the average RMSD values between the predict ligand binding conformations and the native conformations in crystal structures were shown in Table 5. According to the results of cross docking, the average RMSD between the ultimately chosen docking poses and the ligand original poses in crystal structures are all less than 1.5 Å, suggesting molecular docking is accurate in generating the protein-ligand binding conformations for MTases.

**Table 5:** Protein crystal structure structures including the selected structure in cross-docking and the average RMSD between the predict ligand binding conformations and the native conformations.

**Target**	**PDB ID**	**Ligand**	**Average RMSD**	**Selected PDB ID**
CARM1	2y1w	SFG	0.32	2y1w
	5dx1	SFG	0.44	
	5is6	SFG	0.46	
	6arv	SAH	0.46	
	5dwq	SFG	0.48	
	5dxa	SFG	0.49	
	5dxj	SFG	0.49	
	5lv3	SAH	0.50	
	5dx8	SFG	0.56	
	5dx0	SFG	0.62	
	6arj	SAH	1.83	
	2v74	SAH	1.84	
	5ih3	SAH	2.09	
	5u4x	SAH	2.24	
	6d2l	FTG	3.34	
	2y1x	SAH	3.40	
	4ikp	4IK	3.45	
	5k8v	6RE	3.48	
	5is8	SAH	3.59	
	3b3f	SAH	3.65	
DNMT1	3swr	SFG	0.81	3swr
	5gut	SAH	0.89	
	3av5	SAH	0.90	
	3pta	SAH	0.98	
	3pt6	SAH	1.02	
	3pt9	SAH	1.13	
	4wxx	SAH	1.27	
	3av6	SAM	1.32	
	4da4	SAH	2.09	
DOT1L	1nw3	SAM	1.17	1nw3
	3sx0	SX0	1.19	
	4er0	AW1	1.24	
	4ek9	EP4	1.45	
	4ekg	0QJ	1.52	
	5juw	6NR	1.68	
	4eqz	AW0	1.74	
	4hra	EP6	1.74	
	3uwp	5ID	1.76	
	4er7	AW3	1.79	
	4eki	0QK	1.98	
	3qox	SAH	3.68	
	4er3	0QK	3.75	
	3sr4	TT8	3.91	
	3qow	SAM	3.97	
	4er6	AW2	4.03	
	5mw3	5JT	4.19	
	4wvl	3US	4.39	
	4er5	0QK	4.98	
	5mw4	5JU	5.02	
EHMT1	2igq	SAH	0.44	2igq
	3mo2	SAH	0.74	
	3mo5	SAH	0.89	
	3sw9	SFG	0.90	
	4i51	SAH	0.93	
	3fpd	SAH	0.95	
	5tuz	SAM	0.98	
	3hna	SAH	1.01	
	5vsd	SAM	1.11	
	3mo0	SAH	1.13	
	5vsf	SAM	1.15	
	2rfi	SAH	1.18	
	5ttg	SAM	2.49	
	3swc	SAH	2.57	
	5v9j	SAM	2.59	
EHMT2	3k5k	SAH	0.69	3k5k
	5t0m	SAM	0.71	
	5vse	SAM	0.72	
	5tuy	SAM	0.75	
	5v9i	SAM	0.75	
	5jhn	SAM	0.86	
	3rjw	SAH	0.89	
	4nvq	SAH	0.90	
	5t0k	SAM	0.92	
	5jj0	SAM	0.97	
	5jin	SAM	0.98	
	5ttf	SAM	1.02	
	5vsc	SAM	1.04	
	2o8j	SAH	1.14	
	5jiy	SAM	1.73	
SETD8	2bqz	SAH	1.27	2bqz
	1zkk	SAH	1.33	
	3f9z	SAH	1.34	
	5teg	SAM	1.50	
	3f9w	SAH	1.73	
	3f9x	SAH	2.59	
	4ij8	SAM	2.63	
	3f9y	SAH	2.66	
PRMT1	1or8	SAH	0.65	1or8
	1orh	SAH	0.94	
	1ori	SAH	0.97	
	3q7e	SAH	3.99	
PRMT3	1f3l	SAH	0.47	1f3l
	2fyt	SAH	3.95	
PRMT5	5emk	SFG	0.76	5emk
	5emm	SFG	0.91	
	4gqb	0XU	1.15	
	6ckc	F5J	1.29	
	4x63	SAH	1.54	
	5emj	SFG	1.56	
	3ua3	SAH	1.61	
	5c9z	SFG	2.03	
	5eml	SAM	2.10	
	4x60	SFG	2.30	
	5fa5	MTA	2.57	
	4g56	SAH	2.71	
	4x61	SAM	3.13	
PRMT6	4c04	SFG	0.64	4c04
	4y30	SAH	0.70	
	4qqk	37H	0.89	
	5wcf	SAH	0.89	
	4c03	SFG	0.92	
	4hc4	SAH	0.94	
	4c05	SAH	0.99	
	5fqo	SAH	1.24	
	4qpp	SAH	1.29	
	5hzm	SAH	1.29	
	5egs	SAH	1.37	
	4y2h	SAH	1.61	
	5fqn	SAH	3.03	
	5e8r	SAH	3.78	
	4lwp	SAH	3.91	
SETD7	3vv0	KH3	0.72	3vv0
	3vuz	K15	0.91	
	4e47	SAM	1.02	
	4j83	SAM	1.02	
	3m57	SAH	1.05	
	3m5a	SAH	1.07	
	1n6a	SAM	1.14	
	3m55	SAH	1.58	
	3m58	SAH	1.89	
	3cbm	SAH	2.00	
	3cbo	SAH	2.07	
	3m59	SAH	2.36	
	4j7i	SAH	2.38	
	4j7f	SAH	2.41	
	5eg2	SAH	2.46	
	3m53	SAH	2.54	
	4jlg	SAM	2.56	
	3m56	SAH	2.74	
	4j8o	SAH	2.77	
	3cbp	SFG	2.90	
	1o9s	SAH	3.07	
	3os5	SAH	3.07	
	2f69	SAH	3.08	
	3m54	SAH	3.27	
	1xqh	SAH	3.48	
	4jds	SAM	3.73	
	5ayf	SAM	4.31	
	1n6c	SAM	5.91	
	1mt6	SAH	7.82	
SUV39H2	2r3a	SAM	0.69	2r3a

Then, all the small molecules in our dataset are docked into the chosen protein crystal structures in Glide of Maestro version 10.2. Each protein crystal structure was prepared by the Protein Preparation Wizard module of Maestro version 10.2, including adding hydrogens, assigning the bond level, creating disulfide bonds, converting selenomethionines to methionines, and filling in missing side chains using Prime, hydrogen bond network optimization and restrained minimization; removing all the water molecules and metal ions. The protein receptor grids were generated by the Maestro Receptor Grid Generation module of Maestro version 10.2, and the grid centers were set as the centroid of ligands binding in the SAM pocket. All small molecules were prepared by the LigPrep module of Maestro version 10.2, including creating 3D coordinates, calculating ionization states, generating tautomers and stereoisomers, and producing a low energy ring conformation. Grid docking was completed by the Glide module of Maestro Version 10.2, precision of which was set as SP (standard precision) and the number of poses to write out of which was limited to at most 1 per ligand. All other parameters were set as default. Only the binding pose with the best docking score was retained. According to the result of the molecular docking, some molecules preferentially bound other sites than the SAM-binding pocket, and some molecules showed lower docking scores. With the docking score of −8.2 as the threshold, the lower-scored conformations may not be the actual binding conformations, which are not studied in the virtual screening generally. These molecules were disregarded in the followed study. Similarly, binding conformers of decoys were generated through molecular docking by the same process.

#### Interaction Fingerprint Generation

The Fingerprinting Triplets of Interaction Pseudo atoms (TIFP) were used to encode the protein-ligand interaction patterns. Firstly, the interactions between protein and ligand are recognized, including hydrophobic contacts, aromatic interactions, hydrogen bonds, ionic interactions and metal complexation. Then, each interaction was abstract into a pseudo-atom, which is located in the position of the geometric center of the interaction, the acceptor interacted atom or the interacted ligand atom. Then, the number of triples are counted in 6 distance ranges: 0–4, 4–6, 6–9, 9–13, 13–17, 17+Å. Each type of triples is taken as one characteristic and the 211 most common characteristics are retained to form a 211-bit vector.

For each complex crystal structure used for docking, residues within 6 Å of the ligand were retained as binding site information, which was needed for the generation of TIFP fingerprints. The binding sites and selected ligand conformers were converted to the standard mol2 format using chimera (version 1.13). Standard formatted 211-bit TIFPs was generated using IChem software.

#### DNN Model Construction and Evaluation

The DNN model was built by the MultitaskClassifier module of Deepchem (version 2.1.0), and the data set was randomly divided by the RandomSplitter of Deepchem. The Evaluator module of Deepchem was used to evaluate the performance of DNN models.

The evaluation indexed used to evaluate the performance of these modules were area under the precision-recall curve (PRC-AUC) and area under the Compute Receiver operating characteristic curve (ROC-AUC), which are widely used in evaluation the enrichment of scoring model. The closer that the AUC is to 1, the more likely it is that the model is an ideal classification model. Especially, when the ROC-AUC is close to 0.5, the model is close to a random classifier. When the PRC curve reports the evolutions of Recall and Precision, the ROC curve shows the changes of true positive rate (TPR) and false positive rate (FPR):

(1) $$\begin{array}{l} Recall=\frac{N_{TP}}{N_{TP}+N_{FN}} \end{array}$$

(2) $$\begin{array}{l} Precision=\frac{N_{TP}}{N_{TP}+N_{FP}} \end{array}$$

(3) $$\begin{array}{l} \text{TPR}=\frac{N_{TP}}{N_{TP}+N_{FN}} \end{array}$$

(4) $$\begin{array}{l} \text{FPR}=\frac{N_{FP}}{N_{FP}+N_{TN}} \end{array}$$

where N_TP_, N_TN_, N_FP_, and N_FN_ refer to the numbers of true positives, true negatives, false positives, and false negatives, respectively.

The performance of Autodock vina in the test set was also compared with that of DNN model. Before applying Autodock vina (Version 1.1.2), the protein receptor structures and ligand structures were prepared using python scripts named “prepare_receptor4.py” and “prepare_ligand4.py” in AutoDockTools, respectively, which included standard steps such as adding hydrogens and electrons. The grid was also centered on the centroid of the ligand. The grid size was set to 25 Å × 25 Å × 25 Å, and the energy range was set to 4, and all other parameters were used the default settings. The conformation with the best affinity score of each ligand was selected for further study. All figures in this article were produced by Matplotlib and Seaborn python package.

## Conclusions

In this study, we have developed a target-specific classifier for methyltransferases based on protein ligand interaction fingerprint and deep neural network. Binding poses of active and inactive compounds for 12 methyltransferase were generated via molecular docking. TIFP interaction fingerprints were employed as input features of full-connected deep neural network models. The performance of the DNN model on the test set showed that our classifier can classify active and inactive compounds more accurately. In comparison with Glide Autodock vina and DNN-Glide hybrid model, the DNN model improved both classification performance and compound ranking capability.

Currently, the scoring model can be used in virtual screening and experimentally verified. As a target-specific classifier, this neural network model may be applied to other targets through transfer learning, or if the data used for training is appropriate, the classifier of other targets or even the general classifier can be constructed through the same workflow.

## Data Availability

The raw data supporting the conclusions of this manuscript will be made available by the authors, without undue reservation, to any qualified researcher.

## Author Contributions

XLu, WL, and MZ designed research. FL, XWa, JX, XT, XLi, YW, and JZ performed research. XWu, XLiu, and ZL analyzed data. FL, XWa, and MZ wrote the paper.

### Conflict of Interest Statement

The authors declare that the research was conducted in the absence of any commercial or financial relationships that could be construed as a potential conflict of interest.
